# Position statement of the Biotechnology Committee of the Brazilian Society of Rheumatology on the interchangeability of originator and biosimilar biologics in immune-mediated rheumatic diseases

**DOI:** 10.1186/s42358-026-00523-5

**Published:** 2026-01-30

**Authors:** Paulo Chicarone Pereira, Michel Alexandre Yazbek, Rafaela Bicalho Viana Macedo, Fabiana Miranda Moura, Antonio Luíz Boechat, Julio Cesar Bertacini de Moraes, Ricardo Machado Xavier, Hellen Mary da Silveira de Carvalho, Amanda de Miranda Marques, Francisco Airton Castro da Rocha, Fabiana Pompeo de Pina, Carla Gonçalves Schahin Saad, Claudia Diniz Lopes Marques, Karina Rossi Bonfiglioli, Ana Cristina de Medeiros Ribeiro, José Eduardo Martinez, Licia Maria Henrique da Mota, Marco Antonio Araújo da Rocha-Loures, Ivanio Alves Pereira, Claudia Goldenstein Schainberg, Alexandre Ibrahim Uehbe, Valderilio Feijó Azevedo

**Affiliations:** 1 Centro de DoR, São João da Boa Vista, SP Brasil; 2 Universidade Estadual de Campinas, Campinas, SP Brasil; 3 Hospital da Santa Casa de Belo Horizonte, Belo Horizonte-MG, Brasil; 4 Universidade Federal de Minas Gerais, Belo Horizonte, MG Brasil; 5 Universidade Federal do Amazonas, Manaus, AM Brasil; 6 Hospital das clínicas da Faculdade de Medicina da Universidade de São Paulo, São Paulo, SP Brasil; 7 Universidade Federal do Rio Grande do Sul, Porto Alegre, SP Brasil; 8 AutoImune- Unidade de Terapia biológica, Brasilia, DF Brasil; 9 Bio-Manguinhos/ Fiocruz, Rio de Janeiro, RJ Brasil; 10 Universidade Federal do Ceará, Fortaleza, CE Brasil; 11 Universidade Federal de Goiás, Goiania, GO Brasil; 12 Universidade de São Paulo, São Paulo, SP Brasil; 13 Universidade Federal de Pernambuco, Recife, PE Brasil; 14 PontifÍcia Universidade Católica de São Paulo, Sorocaba, SP Brasil; 15 Universidade de Brasília, Brasilia, DF Brasil; 16 Universidade Estadual de Maringá, Maringá, PR Brasil; 17 Universidade do Sul de Santa Catarina, Florianópolis, SC Brasil; 18 NovaClin – terapia assistida em Imunobiológicos, Salvador, BA Brasil; 19 Department of Internal Medicine Edumed, Universidade Federal do Paraná, Rua Pedro Viriato de Souza, 130 - casa 1- Vista Alegre, Curitiba, CEP:80820600 Brasil; 20 Edumed - Centro de pesquisas clínicas, Curitiba, PR Brasil

## Abstract

**Background:**

The increasing availability of biosimilars has raised important questions regarding their interchangeability with originator biologics in the treatment of immune-mediated rheumatic diseases. Addressing this issue is critical to ensuring patient safety, therapeutic efficacy, and informed clinical decision-making.

**Objective:**

To present a position statement from the Biotechnology Committee of the Brazilian Society of Rheumatology on the interchangeability between originator and biosimilar biologic drugs in rheumatologic care.

**Methods:**

A task force of 22 rheumatologists with expertise in immunobiological therapies followed a structured three-phase consensus process: (1) development of five key questions on biosimilar interchangeability; (2) comprehensive literature review using MEDLINE, EMBASE, and LILACS databases; and (3) final review and endorsement by relevant BSR committees. Expert opinion was used when evidence was limited or inconclusive.

**Results:**

The position statement comprises five consensus-based recommendations, all unanimously supported by the task force, and supported by current scientific evidence and clinical experience.

**Conclusion:**

The Biotechnology Committee of the Brazilian Society of Rheumatology endorses the safe and effective interchangeability of originator and biosimilar biologics when guided by principles that ensure patient safety, therapeutic continuity, and healthcare system sustainability.

**Clinical trial number:**

Not applicable.

## Introduction

Biological medicines (or immunobiologics) are large, structurally complex molecules produced by living cells [1, 2]. Owing to their inherent molecular heterogeneity and sensitivity to variations in storage, handling, and manufacturing conditions, it is not possible to produce exact copies of these agents—even during the reproduction of originator biologics themselves [1, 3].

Biosimilars are biological products that exhibit high similarity to an already approved reference biologic in terms of quality, safety, and efficacy, with no clinically meaningful differences [4–6]. In Brazil, biosimilars are approved through the comparability pathway, in alignment with international regulatory standards established by the World Health Organization (WHO), the European Medicines Agency (EMA), and the U.S. Food and Drug Administration (FDA) [6–9].

Establishing a clear and evidence-based position on the interchangeability between biosimilars and originator biologics is essential to reduce uncertainty among healthcare professionals and patients. Moreover, such guidance can support informed prescribing decisions and contribute to expanding access to effective therapies that are critical for disease control and improving the quality of life of individuals with immune-mediated conditions.

## Methods

A task force composed of 22 rheumatologists with recognized clinical and academic expertise in immunobiologic therapies was convened and endorsed by the Brazilian Society of Rheumatology (SBR) to develop this position statement on the interchangeability between originator and biosimilar biologics in immune-mediated rheumatic diseases.

The consensus development process followed a structured, three-phase approach:

### Formulation of key questions

The task force identified and refined five central clinical and regulatory questions related to biosimilar interchangeability. These questions were defined through iterative teleconferences and served as the foundation for the literature review and the development of consensus-based recommendations.

### Literature review

A comprehensive narrative review of the literature was conducted to address the key questions. Searches were performed in the MEDLINE, EMBASE, and LILACS databases using predefined terms, including “biosimilars,” “interchangeability,” “reference products,” “originators,” and “real-world evidence.” Publications were selected based on their methodological robustness and relevance to the Brazilian healthcare context, as determined by expert judgment. In instances where published evidence was limited, inconsistent, or unavailable, recommendations were formulated based on expert consensus from the task force, following a pragmatic, experience-based approach.

### Review and endorsement

The draft statement was reviewed by representatives from the SBR Committees on Rheumatoid Arthritis, Spondyloarthritis, Psoriatic Arthritis, and Public Health Policy. Their input was incorporated into the final version.

## Results

The following questions were asked to taskforce to prepare the recommendations for the prescription of biosimilars. (Table 1)

**Table 1:** Taskforce questions

Question Number	Question
1	Who is the ideal patient to switch from the originator to the biosimilar?
2	What is the minimum period the patient should remain on the same immunobiological before a new switch?
3	Under what circumstances is a reverse switch (return to the originator) justified?
4	Can attending physicians oppose a switch or recommend a return to the originator biologic or another biosimilar?
5	What is the importance of pharmacovigilance and medical education in the context of interchangeability?

**Question 1** - Who is the ideal patient to switch from the originator to the biosimilar?

**Ideal candidates are patients with inactive or low disease activity**,** with effective and well-tolerated treatment with the originator**,** or those initiating therapy with an equivalent biosimilar.**

Ideal candidates for switching from an originator biologic to a biosimilar are patients with inactive disease or low disease activity who are receiving effective and well-tolerated treatment with the originator [10, 11]. Patients initiating therapy with a biosimilar equivalent may also be considered appropriate candidates [12]. In these scenarios, the risk of confounding variables—such as active inflammation, recent treatment modifications, or previous adverse events—is minimized, reducing the likelihood of clinical instability or nocebo responses [10, 13].

This approach is supported by a systematic review and meta-analysis conducted by the U.S. Food and Drug Administration (FDA), which evaluated 44 treatment switching periods (TSPs) involving more than 5,200 patients across 31 studies [14]. The analysis found no statistically significant increase in adverse events, treatment discontinuations, or immunogenicity in patients who were switched to biosimilars compared to those who remained on the originator therapy [14, 15].

In line with these findings, the American College of Rheumatology (ACR) has publicly supported the removal of mandatory switching studies for interchangeability designation. In its 2024 advocacy letter to the FDA, the ACR emphasized that consistent safety and immunogenicity outcomes across studies support the routine use of biosimilars in clinical practice. The organization also highlighted the importance of transparent labeling and full disclosure of data sources to promote prescriber confidence and safeguard patient safety [16].

Evidence from international and Latin American studies reinforces this position. The NOR-SWITCH trial demonstrated non-inferiority in clinical outcomes after switching from infliximab originator to its biosimilar [17]. The PANLAR consensus statement [10] and recent regional data [18], including a Brazilian cohort study [19], also support switching in stable patients. In this study, 78.6% of patients with axial spondyloarthritis maintained remission or low disease activity 12 months after switching from the adalimumab originator to a biosimilar (adalimumab-AACF), with a 94.6% drug retention rate [19].

Although biosimilars approved by stringent regulatory authorities—such as the EMA or those following WHO-aligned pathways—can be considered interchangeable with their reference products, frequent switching within short intervals is not recommended. To preserve pharmacovigilance integrity and minimize confusion among prescribers and patients, current best practices suggest limiting therapeutic switches (either from originators to biosimilars or between biosimilars of the same reference product) to no more than once per year [20].

Conversely, patients with moderate to high disease activity, therapeutic failure, or adverse events related to their current biologic are not suitable candidates for medical switching to a biosimilar. In such cases, switching to a different therapeutic agent with an alternative mechanism of action is preferred [21].

In summary, safe and effective switching requires careful patient selection based on disease stability and treatment tolerability, supported by ongoing pharmacovigilance and transparent regulatory processes.

**Question 2** – What is the minimum period the patient should remain on the same immunobiological before a new switch?

**Multiple switches (between originator and biosimilars or among biosimilars) should be avoided within less than one year.**

The rationale to maintain a minimum 12-month interval between switches of immunobiological agents is based on multiple clinical, pharmacological, and operational considerations. Chief among these are challenges related to immunogenicity assessment, pharmacovigilance and traceability, and the limited availability of robust evidence from studies evaluating short-interval or multiple switches [22, 23].

Immunogenicity, particularly the development of anti-drug antibodies (ADAs), often emerges between 6 and 12 months after initiating biologic therapy. This latency period highlights the need for adequate clinical observation before subsequent therapeutic changes, allowing for a clearer assessment of treatment response and potential adverse events [12, 18, 24].

Frequent switching within shorter time frames may obscure the attribution of efficacy loss or adverse events to a specific product, compromising pharmacovigilance efforts and limiting accurate causality assessment [22, 23]. These concerns are particularly relevant in public health systems with centralized procurement, where multiple non-medical switches may be driven by logistical or financial factors [24].

Moreover, repeated switches within a short period can lead to confusion among patients and healthcare providers, potentially increasing the risk of nocebo effects and reducing treatment adherence. A recent prospective study evaluating reverse switching from biosimilar SB2 to the infliximab originator reported a 15% treatment discontinuation rate, despite clinical stability, likely influenced by negative patient perception [25].

Although high-quality randomized controlled trials such as the NOR-SWITCH study have demonstrated the safety and efficacy of a single switch from originator to biosimilar over a 52-week period, they were not designed to assess multiple or short-interval switches [17]. Similarly, real-world cohort studies (e.g., Danish registries) and systematic reviews suggest no increase in immunogenicity or adverse outcomes with up to two switches, but follow-up durations typically range around 12 months, and data on multiple switches within shorter intervals remain scarce [12, 24, 26].

In addition to clinical concerns, legal and ethical challenges may arise in attributing adverse outcomes to a specific product when multiple switches occur in a short time. These issues underscore the importance of maintaining product traceability, ensuring transparency in clinical decision-making, and preserving the integrity of physician-patient communication.

Taken together, the available evidence and expert consensus support the recommendation to avoid multiple switches within a 12-month period. This precautionary approach promotes therapeutic stability, improves pharmacovigilance accuracy, and enhances patient confidence and adherence.

**Question 3** – Under what circumstances is a reverse switch (return to the originator) justified?

**A reverse switch to the originator should be considered only in exceptional cases of confirmed adverse events or proven loss of efficacy**,** excluding nocebo effects.**

Reverse switching defined as the transition from a biosimilar back to its reference biologic, should be reserved for exceptional clinical circumstances, such as confirmed adverse events or objectively verified loss of therapeutic efficacy. Crucially, such decisions must exclude nocebo-related phenomena, which can mimic treatment failure but are driven by patient perception rather than pharmacological or immunological factors [25, 27].

Although the body of evidence on reverse switching is more limited compared to the extensive literature on originator-to-biosimilar transitions, emerging data support its clinical justification in select scenarios. A prospective study by Fischer et al. evaluated patients with inflammatory bowel disease who underwent a reverse switch from the biosimilar SB2 to reference infliximab. Approximately 14.7% of patients discontinued treatment after the reverse switch; however, no significant changes were observed in objective measures of disease activity or inflammatory markers, suggesting that some discontinuations may have been related to nocebo effects rather than true loss of efficacy or safety concerns [25].

Additional evidence from an open-label extension of the NOR-SWITCH trial demonstrated sustained efficacy and safety of CT-P13 over the long term [17]. Similarly, a multicenter Japanese study involving patients with rheumatoid arthritis in sustained remission following long-term use of infliximab originator reported that most patients maintained disease control after switching to CT-P13. A small subset of patients required switching back to the originator due to perceived loss of efficacy; however, **immunogenicity and pharmacokinetic profiles remained stable, suggesting no pharmacological rationale for the reverse switch [28].

In some cases, medically justified factors may necessitate reverse switching. These include hypersensitivity reactions to biosimilar excipients (e.g., citrate-containing formulations), differences in device usability (e.g., syringe or autoinjector formats), injection volume tolerability, or other patient-specific tolerability concerns. In such instances, returning to the originator is considered a rational therapeutic adjustment, not a nocebo-driven decision [29].

Despite its clinical relevance in selected cases, reverse switching is not consistently addressed in international regulatory frameworks. Nonetheless, expert reviews and consensus statements emphasize that routine or non-medical reverse switching should be discouraged, as it may undermine confidence in biosimilars, disrupt continuity of care, and compromise the sustainability of healthcare systems [29, 30].

Accordingly, in cases of perceived treatment failure or adverse events following a biosimilar switch, a structured clinical reassessment should be the first step. This includes objective evaluation of disease activity (e.g., validated scores and biomarkers), assessment of treatment adherence, and open communication with the patient to explore potential psychological or perceptual contributors. Only when nocebo effects have been excluded and clinical deterioration is confirmed should a reverse switch be considered.

This approach is consistent with best practices in pharmacovigilance and shared decision-making, reinforcing patient trust and supporting the credibility of biosimilars as safe and effective therapeutic alternatives [29].

**Question 4** – Can attending physicians oppose a switch or recommend a return to the originator biologic or another biosimilar?

**Attending physicians may oppose a switch or recommend a return to a previous biologic when clinically justified**,** based on individual patient factors such as disease activity**,** prior therapeutic failure**,** or safety concerns.**

Effective and transparent communication among physicians, patients, and payers is essential to ensure safe and evidence-based decisions regarding the substitution of biologic therapies [31]. While prescriber authorization is not universally mandated for biosimilar substitution, clinicians retain the right to oppose a switch when clinically justified [32]. Objections should be based on patient-specific factors—such as moderate to high disease activity, documented therapeutic failure, adverse events, or immunogenicity concerns—and must be grounded in robust scientific or clinical rationale [29].

Structured mechanisms such as standardized justification forms, appeals boards, or therapeutic committees play a critical role in resolving disagreements and ensuring that substitution decisions are transparent, consistent, and accountable [31]. The prescriber’s clinical judgment remains central, especially in non-medical switching scenarios, where patient safety and therapeutic continuity may be at risk.

Importantly, healthcare professionals must be informed of the specific product dispensed to ensure proper pharmacovigilance, traceability, and continuity of care. The European Medicines Agency (EMA) has emphasized that, even in settings where substitution does not require prior prescriber authorization, maintaining transparency and traceability is non-negotiable [13, 33, 34].

Data from the DANBIO registry in Denmark highlight the clinical impact of excluding physicians from switching decisions. Patients who were automatically switched without individualized reassessment had lower adherence and worse outcomes, reinforcing the need for prescriber involvement in therapeutic transitions, even under system-wide policies [35].

To safeguard patient outcomes, switching decisions should be guided by disease-specific protocols and include comprehensive clinical risk assessments, considering factors such as treatment adherence, disease stability, and prior adverse reactions. A structured governance framework for biosimilar implementation should include:

1. Alignment between prescribers and healthcare administrators on substitution criteria.
2. Routine post-switch monitoring to assess efficacy and tolerability.
3. Transparent communication strategies to address patient perceptions and mitigate nocebo effects.
4. Establishment of multidisciplinary expert committees to resolve conflicts and support clinical decision-making [36].

Although ANVISA does not currently regulate interchangeability, it recognizes biosimilars approved through comparability pathways as therapeutic alternatives [37]. Nonetheless, clinical autonomy must be preserved, particularly in cases where switching may compromise treatment efficacy or safety [38, 39].

In jurisdictions such as the United States, the FDA’s interchangeability designation allows automatic substitution but mandates notification of the prescriber, who retains the authority to oppose future substitutions [40].

In conclusion, when a physician objects to a switch, the rationale must be explicitly documented and based on sound clinical reasoning. Preserving the integrity of the physician-patient relationship and supporting shared decision-making are essential to maintaining confidence in biosimilars and ensuring responsible integration into clinical practice.

**Question 5** - What is the importance of pharmacovigilance and medical education in the context of interchangeability?

**Pharmacovigilance and structured medical education are essential pillars for the safe and effective implementation of biosimilar interchangeability in clinical practice. These elements not only ensure alignment with regulatory standards but also play a critical role in safeguarding patient safety**,** maintaining therapeutic continuity**,** and fostering public and professional confidence in the adoption of biosimilars.**

The American College of Rheumatology (ACR) underscores the critical role of a robust pharmacovigilance infrastructure—encompassing mandatory reporting systems and integration with disease-specific registries—as a prerequisite for the safe and effective adoption of biosimilars in rheumatology [41]. This is especially important in the context of chronic immune-mediated diseases, where long-term safety monitoring and the identification of immunogenicity patterns depend on precise and sustained data collection [31].

Establishing such a pharmacovigilance framework requires comprehensive clinical governance, including standardized institutional protocols for adverse event reporting, systematic assessment of therapeutic outcomes following biosimilar transitions, and efficient communication channels between prescribers and healthcare administrators. The formation of multidisciplinary scientific-technical committees is also recommended to mediate disputes and ensure alignment with best clinical practices [36]. These governance strategies promote consistency in clinical decision-making, enhance system accountability, and help build sustained trust among clinicians and patients.

Beyond ensuring safety, pharmacovigilance serves a broader role in strengthening the credibility and transparency of health systems. By capturing real-world effectiveness and safety data, pharmacovigilance reinforces prescriber and patient confidence—particularly in settings with limited prior exposure to biosimilars. Moreover, continuous monitoring enables evidence-based updates to clinical guidelines, regulatory policies, and reimbursement frameworks, thereby contributing to the long-term sustainability of biosimilar integration [42].

Complementing pharmacovigilance, structured medical education—targeted at both healthcare professionals and patients—is essential to closing knowledge gaps regarding biosimilar development, regulatory approval, and clinical equivalence. Leading international organizations, including EULAR, ACR, and PANLAR, consistently advocate for ongoing educational initiatives to support informed decision-making and reduce misconceptions [10, 13, 41, 43]. Adequately informed clinicians are better equipped to engage in shared decision-making, address patient concerns, and avoid misattribution of adverse outcomes to the biosimilar itself.

In parallel, patient-centered education plays a key role in mitigating the nocebo effect, a well-documented contributor to treatment discontinuation. For instance, Fischer et al. reported that some patients with inflammatory bowel disease who reverted to the originator infliximab after a biosimilar switch cited subjective loss of efficacy, despite stable objective disease measures [44]. These findings emphasize the influence of psychological factors on treatment perception and the importance of educational reassurance in maintaining adherence.

Together, pharmacovigilance and medical education provide a synergistic foundation for the successful integration of biosimilars. Their implementation ensures that interchangeability is conducted in a scientifically sound, transparent, and patient-centered manner—ultimately preserving therapeutic integrity and optimizing outcomes across healthcare systems.

The five recommendations for prescription of biosimilars based on each question are summarized in the Table 2.

**Table 2:** Recommendations for the prescription of biosimilars

Recommendation	Description
1. Ideal Candidates for Switching	Ideal candidates are patients with inactive or low disease activity, with effective and well-tolerated treatment with the originator, or those initiating therapy with an equivalent biosimilar.
2. Avoiding Multiple Switches	Multiple switches (between originator and biosimilars or among biosimilars) should be avoided within less than one year.
3. Reverse Switching	A reverse switch to the originator should be considered only in exceptional cases of confirmed adverse events or proven loss of efficacy, excluding nocebo effects.
4. Prescriber Awareness	Attending physicians may oppose a switch or recommend a return to a previous biologic when clinically justified, based on individual patient factors such as disease activity, prior therapeutic failure, or safety concerns.
5. Pharmacovigilance and Medical Education	Pharmacovigilance and structured medical education are essential pillars for the safe and effective implementation of biosimilar interchangeability in clinical practice. These elements not only ensure alignment with regulatory standards but also play a critical role in safeguarding patient safety, maintaining therapeutic continuity, and fostering public and professional confidence in the adoption of biosimilars.

## Discussion

Brazil currently ranks as the second-largest public market for biosimilars worldwide, surpassed only by the European Union [45]. Despite important regulatory advances, the absence of nationally standardized criteria for interchangeability introduces variability in clinical practice and uncertainty among healthcare professionals. This position statement reinforces that biosimilar transitions, when performed under well-defined conditions—such as disease stability, transparent physician–patient communication, and robust pharmacovigilance—do not compromise efficacy or safety.

Consistent evidence from randomized controlled trials, including the NOR-SWITCH study [17], and meta-analyses [14] has demonstrated no significant differences in clinical outcomes or immunogenicity between originator biologics and their biosimilar counterparts [46–48]. These findings support the clinical rationale for interchangeability when grounded in evidence-based protocols.

Importantly, health systems that implement structured and transparent interchangeability policies can expand access to biologic therapies while maintaining therapeutic standards. Economic assessments in the Brazilian private healthcare context demonstrate substantial cost savings through structured biosimilar switch programs—even outside the public system [18].

Educational strategies targeting both prescribers and patients are also essential. Improving understanding of biosimilar development, regulatory approval processes, and real-world evidence can mitigate nocebo effects, enhance treatment adherence, and promote confidence in biosimilar therapies [10, 13, 29].

Overall, this position statement provides a framework for clinical governance and public health policy, promoting the responsible adoption of biosimilars based on scientific evidence, ethical principles, and a commitment to patient-centered care.

### Limitations

Although a comprehensive review of the literature was conducted, evidence regarding multiple biosimilar switches, particularly within the field of rheumatology, remains limited. Therefore, this position statement reflects the current expert interpretation of available data and may require updates as new evidence emerges.

## Conclusion

The interchangeability between originator and biosimilar biologics is a key component in broadening patient access to effective treatments and promoting the sustainability of healthcare systems. The Biotechnology Committee of the Brazilian Society of Rheumatology supports the use of biosimilars, provided that their implementation is guided by principles that ensure patient safety, clinical efficacy, transparency, and continuity of care.that guarantee patient safety, clinical efficacy, and continuity of care.

## Data Availability

The datasets during and/or analysed during the current study available from the corresponding author on reasonable request.
